# Precision in Motion Management: Long-Term Local Control and Prognostic Insights in SBRT for Oligometastatic Lung and Liver Metastases

**DOI:** 10.3390/cancers17020296

**Published:** 2025-01-17

**Authors:** Silke Dirkx, Sven Van Laere, Thierry Gevaert, Mark De Ridder

**Affiliations:** Department of Radiotherapy, Research Centre for Digital Medicine, VUB-UZ Brussel, 1090 Brussels, Belgium

**Keywords:** oligometastases, stereotactic body radiotherapy, long-term local control, motion management, prognostic factors

## Abstract

**Background/Objectives**: Inadequate dosing and respiratory motion contribute to local recurrence for oligometastatic disease (OMD). While short-term LC rates are well-documented, data on long-term LC remain limited. This study investigated long-term LC after stereotactic body radiotherapy (SBRT), using respiratory motion management techniques. **Methods:** This retrospective study took place at UZ Brussel with follow-up until Oct 2024. It analyzed oligometastatic patients treated with SBRT between Jul 2012 and Feb 2017. Treatment involved delivering 50 Gy in 10 fractions on the 80% isodose line, building on data from a prior prospective study. Lesion movement was managed using internal target volume (ITV) or dynamic tumor tracking (DTT) with marker. The primary endpoint of the study was long-term LC and identifying variables associated with it using a Cox proportional hazards model. **Results**: A total of 100 patients were treated for a total of 211 metastatic lesions. Lesions were predominantly in the lungs (74%) and treated using ITV (88%). LC rates at 1, 3, 5, and 10 years were 76.5%, 53.8%, 38.1%, and 36.3%, respectively. Improved LC was observed in locations other than lung and liver (HR: 0.309; *p* = 0.024) and with increasing age (HR: 0.975; *p* < 0.010). Worse LC was seen in liver lesions (HR: 1.808; *p* = 0.103) and systemic therapy post-radiotherapy (HR: 3.726; *p* < 0.001). No significant associations were found with tumor size or tumor motion, nor between the two motion management strategies used (DTT and ITV). **Conclusions**: Appropriate motion management is key in LC for OMD. No significant difference in LC was found between both techniques. Lesion location, patient age, and systemic therapy post-radiotherapy were prognostic factors for LC.

## Simple Summary

Inadequate dose prescription and inadequate respiratory motion management contribute to local recurrence in short-term studies of oligometastatic disease. This study investigated long-term local control (LC) after Stereotactic Body Radiotherapy (SBRT), using respiratory motion management techniques such as internal target volume or dynamic tumor tracking. A total of 50 Gy was delivered in 10 fractions (80% isodose line). The aim of our study was long-term LC and its predictive factors. Progression-free survival (PFS) and overall survival (OS) were secondary endpoint. A total of 211 Metastatic lesions in 100 patients were treated. Lesions were predominantly in lungs (74%) and treated using ITV (88%). LC rates at 1, 3, 5, and 10 years were 76.5%, 53.8%, 38.1%, and 36.3%, respectively. Improved LC was seen in locations other than lung and liver and with increasing age. Worse LC was seen in liver lesions and systemic therapy post-radiotherapy. PFS rates at 1, 3, 5, and 10 years were 34.7%, 18.2%, 14.4%, and 9.8%, respectively. OS rates at 1, 3, 5, and 10 years were 85.1%, 59.3%, 32.0%, and 9.6%, respectively.

## 1. Introduction

More than half of the patients with solid tumors succumb because of a malignancy with distant metastases. Oligometastatic disease (OMD) is defined by ESTRO-ASTRO as a disease with 1–5 metastatic lesions, with or without a controlled primary tumor [1,2].

In patients with OMD, local treatment as surgical resection shows good results in local control (LC) [3,4,5,6,7]. In comparison with surgery, stereotactic body radiation therapy (SBRT) is considered a good alternative for local treatment in oligometastatic cancer patients, with a promising LC, toxicity, and survival outcome [3,4,8,9]. SBRT is defined by the American Society of Radiation Oncology (ASTRO) as a treatment with a relatively high dose of radiation in a few fractions, which results in a high biological effective dose (typically more than 100 Gy) [1,5,10]. In the past, thoracic and high-abdominal radiotherapy was limited by the toxicity of normal tissue, due to respiratory motion, which is a significant factor for geometric and dosimetric uncertainties during treatment planning and delivery in [11,12,13].

Previous studies in our hospital studied short-term LC in OMD with lung and liver lesions with an irradiation dose in 10 fractions.

In a first phase II trial, homogeneously, 40 Gy was delivered in 10 fractions with an insufficient 1-year LC of 54% [14]. In a second follow-up study, 50 Gy was prescribed without a significant improvement of 1-year LC (54%) [15]. Further analysis of the recurrences showed both in-field and marginal recurrences [16]. The in-field recurrences could be explained by the relatively low irradiation dose (BED_(α/β=10)_ of 56 and 75 Gy, respectively). The marginal recurrences were associated with positioning inaccuracies as moving lesions had a significantly higher failure risk. The third trial prospectively analyzed 1-year LC with proper respiratory management (internal target volume (ITV) and dynamic tumor tracking (DTT)) and dose escalation (50 Gy/10 fractions on the 80% isodose line). The 1-year LC increased to 89%.

In most irradiations, a planning target volume (PTV) is designed to assure optimal target coverage and take inter- and intra-fractional errors (variations of tissue positions, sizes, and shapes, as well as variations in patient position and beam geometries) into account [17]. Without motion management, this margin will be large and will be the limiting factor for safe irradiation. That is why it is important to compensate for respiratory motion. The advances of technology made it possible to delineate/define more accurately the location of the tumor. The PTV margin can be reduced by controlling the inter- and intra-fraction tumor motion, for example, by the use of ITV, tracking, or deep inspiration breath hold techniques [18,19].

In addition to lung metastases, the liver is a frequent site of metastases for various primary tumors. Surgical resection remains the main treatment for operable patients with resectable liver metastases from, e.g., colorectal cancer. When the patient is inoperable, local treatment, such as radiofrequency ablation (RFA), microwave ablation (MWA), or SBRT, is an appropriate alternative [20].

The aim of this study was to investigate long-term LC after SBRT, using respiratory motion management techniques, ITV, and tracking, examining their prognostic factors as well as the long-term progression-free survival (PFS) and OS.

## 2. Materials and Methods

### 2.1. Patient Inclusion

The study included adult patients with oligometastatic cancer, treated in a single university center (UZ Brussel), characterized by 1–5 metastatic lesions from any primary origin and an Eastern Cooperative Oncology Group (ECOG) performance status of ≤2. To qualify, the primary tumor must have been treated with curative intent using methods such as surgery, radiotherapy, or chemoradiotherapy, and systemic treatment must have been completed 1 month prior to the SBRT. For cases involving liver metastases, patients were required to have a functional liver volume exceeding 1000 cc, while those with lung metastases needed a lung diffusion capacity for carbon monoxide (DLCO) of over 30%. Patients were excluded if they had contraindications for radiation, such as violations of OAR constraints, metastases from another carcinoma, or Child-Pugh B or C liver cirrhosis. This study represents a retrospective continuation of a previously conducted prospective study on short-term local control (LC) at our center [16]. Informed consent was obtained from all patients at that time (ClinicalTrials.gov NCT02228356).

### 2.2. Study Design

This study aimed to assess long-term LC in patients with oligometastatic disease treated with SBRT, using motion management techniques. The treatment period spanned from July 2012 to February 2017, with follow-up until October 2024.

The primary objective was to evaluate the 1-, 3-, 5-, and 10-year LC. As a secondary objective, predictors of local recurrence were analyzed, as was the 1-, 3-, 5-, and 10-year PFS and OS. In the analysis of the predictive factors, the following factors where included: gender, age, ECOG, year of radiotherapy, synchronous/metachronous, nodal involvement, primary location (colorectal carcinoma/non-colorectal carcinoma), histology, intention to treat, location, brain metastasis, previously received systemic therapy, afterwards systemic therapy, GTV (in cc), and ITV–GTV ratio. GTV is used as a size parameter, as the ITV–GTV ratio is a parameter used to represent the level of motion of a lesion. For the tracking method, the ITV was defined afterwards, to make a comparison between the two groups. The location of SBRT for the metastatic cancer tumor was regrouped into lung lesions, liver lesions, and others. The category ‘others’ included pleural, subcutaneous, nodular, renal, adrenal, and glandular lesions.

### 2.3. Motion-Management Techniques

Patients were positioned in supine position, when a planning 4DCT (to assess breathing motion) was performed. In metastases with a peak-to-peak amplitude (PPA) ≥ 7 mm, an internal marker was placed into the metastasis, with a maximum of 3 markers per patient. Prior assessments demonstrated that coil migration did not occur [21,22]. Those lesions were treated with dynamic tracking. If the PPA is <7 mm or the placement of a marker was contra-indicated, the ITV-method was used, by blending the GTV delineations on all 10 breathing phases of the 4DCT. A daily cone beam CT imaging was performed, positioning was based on soft tissue verification. If the lesions displayed a PPA ≥ 7 mm in any direction, and if there was no contra-indication, an internal marker was placed into the metastasis with the largest PPA. The PTV has a margin of 5 mm around the GTV with the use of the tracking method, or around the ITV in other cases. A larger PTV margin of 8 mm was used in lesions smaller than 1 cm, as they are more difficult to visualize on the CBCT. All lesions (DTT or ITV method) were treated with the VERO SBRT 6MV Linac (collaborative product between Mitsubishi Industries Ltd. and Brainlab AG, respectively Tokyo, Japan and Munchen, Germany). Conformal beam radiotherapy was used to treat all lesions with DTT. In the case of ITV, conformal beam or static field intensity-modulated radiotherapy (IMRT) was used depending on lesion location and/or OAR.

Patient Specific Quality Assurance (PSQA) was applied for all patients using the MapCheck (Sun Nuclear, Melbourne, FL, USA). For the moving targets, dynamic tracking was verified using QUASAR phantom (IBA Dosimetry, Schwarzenbruck, Germany) on a monthly basis.

### 2.4. Treatment Schedule

All patients were treated in 10 daily fractions, with a total dose of 50 Gy, prescribed on the 80% isodose line (exceptionally individualized based on toxicity or need for dose escalation to the 60% or 100% isodose line). The 100% isodose line (6.25 Gy/fraction, BED_(α/β=10)_ = 101.6 Gy) had to encompass at least 60% of the GTV or ITV with a heterogeneous dose profile. The maximum dose delivered to the central tumor core did not exceed 6.9 Gy/fraction (110% of 6.25 Gy). Dose constraints are summarized in Table 1.

### 2.5. Assessment

Local response and new lesions were defined using RECIST (Response Evaluation Criteria in Solid Tumors) or PERCIST (Positron Emission Tomography Response Criteria in Solid Tumors) on CT or PET-CT imaging, respectively, performed at approximately 3-month intervals.

### 2.6. Statistical Analysis

Descriptive statistics were summarized using medians and interquartile ranges (Q1–Q3) for continuous variables, while categorical variables were reported as counts and percentages. LC, PFS, and OS rates were estimated using the Kaplan–Meier method. Median survival was estimated for LC, PFS, and OS. Predictors of local recurrence were analyzed using Cox proportional hazards, with model selection based on AIC in both directions. To adjust the analysis for bigger or smaller tumors on the one hand and for lesions with less or more movement on the other hand, we, by default, chose to adjust for tumor size and tumor movement, respectively, using the GTV and ITV–GTV ratio as described above. All volumes in the analysis were measured in cubic centimeters (cc). The assumption of proportional hazards was tested for each variable in the Cox proportional hazards model; variables violating this assumption were later removed from the analysis. Grade 3 or higher toxicities were recorded using the Common Terminology Criteria for Adverse Events (CTCAE) version 5.0. To minimize bias inherent to the retrospective design, this analysis was limited to patients who were directly followed up at UZ Brussel and had not been referred back to their original referring institutions. All analyses were performed using the statistical software R running within RStudio. For statistical inference, a significance level of α = 0.05 was applied throughout the study.

## 3. Results

### 3.1. Patient Sample

A total of 211 metastatic lesions in 100 patients were treated with SBRT for this analysis. The patient characteristics are shown in Table 2. The median age was 67 years old, with a male predominance (63%). Adenocarcinoma was the most common histology of the primary tumor (65%). More than half of the patients (52%) had a colorectal carcinoma as primary tumor location. The details of the primary tumor locations are summarized in Appendix A, Table A1. Brain metastases were present in 24% of the patients.

Lesion characteristics were summarized in Table 3. The majority of the lesions were present in the lung (74%). CRC accounted for 89% (*n* = 134) of the adenocarcinomas. A total of 96% of the lesions received a dose of 10 × 5 Gy on the 80% isodose, 4% on the 100% isodose, and <1% on the 60% isodose. A total of 89% of the lesions were treated using the ITV-method; the others were treated using marker tracking. The lesions had a median GTV tumor size of 2.5 cc, with a larger volume in lesions treated with tracking (median of 11.3 cc). The median ITV–GTV volume of all lesions was 2.2 cc, with a bigger ‘hypothetical’ volume (= movement) in the tracking group (median of 7.8 cc). Systemic treatment was administered before SBRT in 85% of the patients and afterwards in 39% of the patients. The details of systemic therapy were summarized in Appendix A, Table A2.

### 3.2. Long-Term Local Control and Risk Factor Analysis

**Figure 1:**
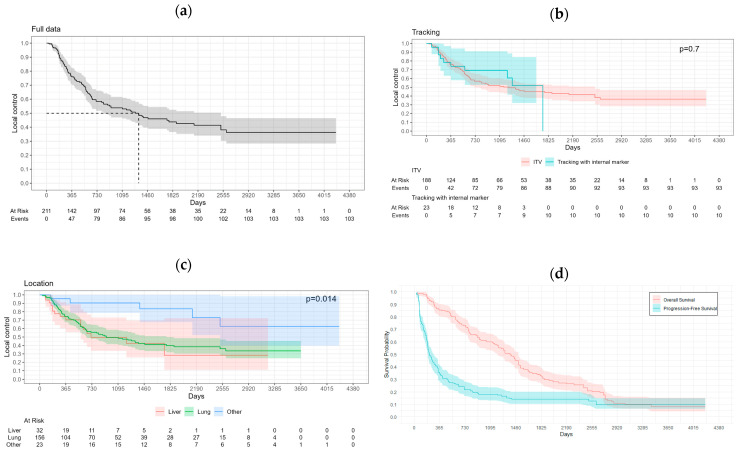
Kaplan–Meier curves. (**a**) Global local control (LC) curve (solid line) with confidence interval. The dashed line represents the median survival time for LC. (**b**) LC according to motion management technique. (**c**) LC according to lesion location. (**d**) Progression-free survival and overall survival.

LC at 1, 3, 5, and 10 years was, respectively, 76.5%, 53.8%, 38.1%, and 36.3%. Median LC was observed at 1332 days (about 44 months). The Kaplan–Meier curves for LC are shown in Figure 1.

The multivariable Cox proportional hazards model (Table 4) showed that lesions in the liver had an 80% higher risk of local recurrence compared to those in the lungs (HR = 1.808; 95% CI: 0.888–3.682; *p* = 0.103). On the other hand, tumors located in ‘other locations’ showed a 70% lower risk of local recurrence (HR = 0.309; 95% CI: 0.112–0.857; *p* = 0.024). Each year of patient age was associated with a 3% reduction in the risk of local recurrence (HR = 0.975; 95% CI: 0.956–0.994; *p* = 0.010). Patients who received systemic therapy after radiotherapy had a 2.7-times higher risk of local recurrence (HR = 3.726; 95% CI: 2.057–6.750; *p* < 0.001).

No significant associations were found with the covariables tumor size (expressed in terms of GTV volume (in cc)) and motion management (expressed in terms of the ratio between GTV and ITV volume). Per cc increase in GTV, the risk for local recurrence decreased by, on average, 0.2% (HR: 0.998; 95% CI: 0.976–1.019; *p* = 0.820). When the ratio between ITV and GTV increased by one unit, we observed a decrease in risk by about 4.3% (HR: 0.957; 0.852–1.077; *p* = 0.428). Both effects were considered not statistically significant.

PFS at 1, 3, 5, and 10 years was, respectively, 34.8%, 18.2%, 14.4%, and 9.9%. The median PFS was 217 days (about 7 months). Overall survival at 1, 3, 5, and 10 years was, respectively, 85.1%, 59.3%, 32.0%, and 9.6%. The median OS was 1386 days (about 46 months or 3 years and 10 months).

The toxicities observed within the first year were well managed. Among the patients who were followed up at our center, only one out of 62 patients (with 137 lesions) (2%) experienced grade 3 toxicity, specifically, radiopneumonitis, within one year following SBRT.

## 4. Discussion

We report the outcome of 211 metastases in 100 patients after SBRT, using respiratory motion management techniques such as ITV or tracking. LC at 1, 3, 5, and 10 years was, respectively, 76.5%, 53.8%, 38.1%, and 36.3%. The one-year control rate is lower than indicated in the previous trial of SBRT in oligometastatic disease in our center (89%). This might be due to a higher percentual rate of salvage SBRT as intention to treat (and, so, more resistant tumors) instead of SBRT treatment as primary choice, being 20% compared to 51% in our study [16].

The tracked lesions had a larger average size compared to ITV lesions. LC treated with ITV methods was higher than that treated with tracking; however, a significant difference could not be determined. This means that the system was able to track the tumor movements accurately and that LC can be maintained regardless of the motion amplitude.

Most of the SBRT schedules use less fractions. Prior SBRT studies with less fractions showed a short-term local control of 70% or higher, although with a major difference in dose, target location, and histology [4,23,24,25,26,27]. Fewer trials described a 5-year LC. Stera et al. and Yamamoto et al. described a 5-year LC of 69% or higher in lung or liver metastasis, with a median BED_(α/β=10)_ of 125 Gy. Despite of the higher LC, both studies noted a higher toxicity in grade 3, but also grade 4 and even grade 5 toxicity. This might be due to the high irradiation dose and the absence of motion management techniques [28,29]. A reason for lower LC in our study can be the inclusion of resistant tumors, since more than 50% of our metastases were treated with salvage radiotherapy. Although the intention to treat was not traceable in the abovementioned studies, no formal comparison could be made. To the best of our knowledge, this trial is the only one to analyze 10-year LC.

This 10-fraction schedule shows an advantage in treating lesions near important organs at risk (such as heart, bronchi, and bowels) as the grade 3 toxicity was relatively low, and there were no higher grades of toxicity noted.

The multivariable analysis showed that the LC was significantly associated with the location of the metastasis, age, and receiving systemic therapy post-radiotherapy. An interesting correlation with LC was obtained with the variable of receiving systemic therapy after SBRT. The patients who received systemic therapy following SBRT likely had more aggressive disease at the time of SBRT, which may partially explain the observed outcome. Systemic therapies are typically administered in progressive or disseminated metastatic disease, which could indicate a more advanced or resistant disease state. As a result, patients who require systemic treatment after radiotherapy may already have a worse prognosis at baseline, which could contribute to the poorer LC rates observed in this group. Related to this, the previous, short-term study in our center showed that patients resistant to systemic therapy appear less good candidates for SBRT, most likely caused by the cross-resistance mechanisms and larger viable tumor load [16,30].

The explanation for a better long-term LC in locations other than lung and liver may be the tumor biology. Tumors in the “other” group may exhibit less aggressive metastatic behavior or may be less likely to develop radioresistance compared to lung or liver metastases [31]. Renal, adrenal, and glandular lesions may be less prone to hypoxia or vascular issues, which could make them more responsive to radiation [32,33]. More than half of the ‘other’ lesions are nodular, as this is a less advanced disease compared to metastases in the lung or liver [34].

The model results showed that long-term LC was not associated with tumor size (in terms of GTV volume) and tumor motion (in terms of the ITV–GTV ratio). Despite the lack of statistical significance in HRs and their 95% CI, a trend was observed: For larger tumors, the HR decreased by only 0.2% on average per 1 cc increase in GTV, and for less mobile tumors, the HR declined by 4.3% on average per unit increase in the ITV–GTV ratio. These findings suggest that motion management may provide comparable or even slightly improved LC for tumors with greater motion compared to those with less mobility, when using this proposed technique of tumor tracking. However, these differences were statistically not significant.

The result of the median PFS rate in our study (7 months) was in line with the expected rates of previous studies. Baschnagel et al. and Stera et al. observed, respectively, a median PFS of 5.0 and 6.7 months [4,28].

Short-term OS analyzed in our trial was comparable with that in other trials [4,28,29], although the results of long-term OS (>5 years FU) were not yet published, to the best of our knowledge. De Vin et al. defined a 5-year OS rate of 19%, in comparison to 32% in our study [35]. This was lower due to the radiation dose (median BED_(α/β=10)_ = 61 Gy) and the inclusion of intracranial lesions, which have, in general, a worse prognosis.

No statistically significant difference was observed between the two motion management techniques used in our study (ITV and DTT). This lack of significance is likely attributed to the relatively small sample size of lesions treated with the tracking method, as well as the selection strategy employed. It is also possible that there is, in fact, no detectable difference between the two approaches. This study has some limitations that should be considered for future work. A relatively small proportion of liver metastases (15% of the total lesions) were included in this analysis. This is likely due to the often-used radiofrequency ablation (RFA) procedure for liver metastasis management at our institution. As these lesions have a different biological behavior and clinical course, the inclusion of more liver metastases in future studies would provide a better understanding of SBRT efficacy in this context.

Due to the retrospective design, it was not possible to evaluate radiotherapy-related toxicity for patients who had follow-up in their referring institutions. Toxicity data, including late effects of SBRT, were, therefore, not fully available. In addition, this study was conducted at a single institution, and the results may not be generalizable to other centers with different patient populations, treatment protocols, or technologies. Multicenter, larger-scale studies would provide a broader perspective on the effectiveness of SBRT for OMD.

## 5. Conclusions

Local control after 1, 3, 5, and 10 years is, respectively, 76.5%, 53.8%, 38.1%, and 36.3%. With proper motion management, the LC of static as well as movable oligometastatic lesions can be maintained. Factors such as the location of the lesion, patient age, and administration of systemic therapy after SBRT have important roles in the therapeutic outcome of OMD, and can help to delay the time for the (re)start of systemic therapy.

## Figures and Tables

**Table 1:** Dose constraints organs at risk.

Organ	Parameter	Constraint
Lungs	V20	≤25%
Liver (whole liver—GTV)	V30	≤30%
Liver (whole liver—GTV)	Median dose	≤22 Gy
Spinal cord	Maximum dose	≤36 Gy
Kidney (ipsilateral)	V21	≤30%
Kidney (contralateral)	V21	≤5%
Oesophagus, stomach, bowels	Maximum dose	≤40 Gy

**Table 2:** Patient characteristics (*n* = 100).

	*n* = 100 (100%)
Demographics	
Age (years)	
Median	67
Q1–Q3	58–74
Gender (*n*, %)	
Male	63 (63)
Female	37 (37)
ECOG (*n*, %)	
0	25 (25)
1	67 (67)
2	8 (8)
Histology (*n*, %)	
Adenocarcinoma	65 (65)
Spinocellular carcinoma	20 (20)
Others	15 (15)
Primary tumor (*n*, %)	
Colorectal carcinoma (CRC)	52 (52)
Non-colorectal carcinoma (nCRC)	48 (48)
Timing (*n*, %)	
Synchronous oligometastatic disease	10 (10)
Metachronous oligometastatic disease	90 (90)

Abbreviations: ECOG 0 = fully active, no performance restrictions; ECOG 1 = strenuous physical activity restricted, fully ambulatory and able to carry out light work; ECOG 2 = capable of all self-care but unable to carry out any work activities, up and about >50% of waking hours. ‘Other primary tumors’ includes melanoma, clear cell kidney carcinoma, poorly differentiated or undifferentiated carcinoma, cholangiocarcinoma, transitional cell carcinoma, sarcoma, ductal carcinoma, and follicular carcinoma.

**Table 3:** Lesion characteristics (*n* = 211).

	*n* = 211 (100%)
Location of the lesion (*n*, %)	
Lung	156 (74)
Liver	32 (15)
Others	23 (11)
Radiation dose 10 × 5 Gy (*n*, %)	
60% isodose line	1 (<1)
80% isodose line	202 (96)
100% isodose line	8 (4)
Intention of radiotherapy (*n*, %)	
SBRT primary choice	64 (31)
Consolidation	41 (20)
Salvage	106 (51)
Method of treatment	
Tracking	23 (11)
ITV	188 (89)
Tumor size in terms of GTV (cc) (median, Q1–Q3)	2.5 (1.1–9.2)
Tracking	11.3 (4.0–22.3)
ITV	2.1 (1.0–6.5)
Tumor movement in terms of ITV-GTV ratio (median, Q1–Q3)	2.0 (1.5–2.8)
Tracking	1.7 (1.5–2.4)
ITV	2.0 (1.5–2.8)

Abbreviations: Gy (Gray); SBRT (stereotactic body radiotherapy); GTV (gross tumor volume); ITV (internal target volume).

**Table 4:** Cox proportional hazards model for local control (*n* = 211 lesions).

	N	%	HR	Lower Bound	Upper Bound	*p*-Value ^2^
Tumor size (GTV, in cc)			0.998	0.976	1.019	0.820
Tumor movement (ITV–GTV ratio)			0.957	0.852	1.077	0.428
Age (in years)			0.975	0.956	0.994	**0.010**
Location						**0.004**
Lung	156	74	Ref.			Ref.
Liver	32	15	1.808	0.888	3.682	0.103
Others ^1^	23	11	0.309	0.112	0.857	**0.024**
Systemic therapy post-radiotherapy	137	65	3.726	2.057	6.750	**<0.001**
Motion management technique						0.128
ITV	188	89	Ref.			
Tracking	23	11	0.573	0.269	1.221	0.128

^1^ Other locations than lung and liver, here, refer to pleural, subcutaneous, nodular, renal, adrenal, and glandular lesions. ^2^ *p*-values for overall variable effects were right-aligned in the ‘*p*-value’ column. *p*-values that indicate the significance of each category compared to the reference level were left-aligned in the ‘*p*-value’ column. *p*-values in bold indicate statistically significant values. Abbreviations: HR (hazard ratio); GTV (gross tumor volume); ITV (internal target volume). Other factors analyzed but not conducted as significant predictor for local recurrence include gender, age, ECOG, year of radiotherapy, synchronous/metachronous, nodal involvement, primary location (CRC-nCRC), histology, intention to treat, previously received chemotherapy or targeted therapy, afterwards immunotherapy, and targeted therapy.

## Data Availability

The data presented in this study are available upon request from the corresponding author, if asked within a reasonable time frame.

## Appendix A

**Table A1:** Primary tumor of 211 lesions.

Primary Tumor Location	*n* = 211 (100%)
Colo/rectosigmoïd	134 (64)
Head and neck	23 (11)
Lung	16 (8)
Oesophagus	14 (7)
Breast	8 (4)
Melanoma	6 (3)
Kidney	3 (1)
Bile duct	2 (<1)
Bladder	1 (<1)
Endometrium	1 (<1)
Soft tissue (sarcoma)	1 (<1)
Prostate	1 (<1)
Thyroid	1 (<1)

**Table A2:** Descriptive analysis of all 211 lesions.

	*n* = 211 (100%)
Systemic therapy prior to SBRT (*n*, %)	
Yes	179 (85)
No	32 (15)
Chemotherapy (*n*, %)	
Yes	174 (82)
1° line	102 (48)
2° line	43 (20)
3° line	12 (6)
4° line	10 (5)
5° line	7 (3)
No	37 (18)
Immunotherapy (*n*, %)	
Yes	82 (39)
No	129 (61)
Targeted therapy (*n*, %)	
Yes	6 (3)
No	205 (97)
Systemic therapy after SBRT (*n*, %)	
Yes	137 (65)
No	63 (30)
NA	11 (5)
Chemotherapy (*n*, %)	
Yes	128 (61)
1° line	69 (33)
2° line	22 (10)
3° line	27 (13)
4° line	10 (5)
No	72 (34)
NA	11 (5)
Immunotherapy (*n*, %)	
Yes	103 (49)
No	97 (46)
NA	11 (5)
Targeted therapy (*n*, %)	
Yes	41 (19)
No	159 (75)
NA	11 (5)

Abbreviations: NA (not available).
